# Extra Supplement—The Nursing Mirror

**Published:** 1890-09-13

**Authors:** 


Being the Extra Nursing Supplement of "The Hospital" Newspaper.

All Contributions for this Supplement should be addressed to the Editor, The Hospital, 140, Strand, London, W.C., and should have the word "Nursing" plainly written in left-hand top corner of the envelope.

### En Passant.

**PROMPTITUDE.**—The following account from the "Recollections of a Nurse" of how rapidly the writer, in 1879, responded to the invitation of the Baroness Burdett-Coutts, is a lesson to all nurses: "The Baroness, at the head of a ladies' committee, was anxious to send nurses and medical comforts to our sick and wounded soldiers in South Africa. At 6 p.m. it was decided that I should go as the superintendent, and a list of things was given to me that the ladies' committee wished us each to have. Early the next morning I with two others went to Silver's, in Cornhill, and got our things. At 1 p.m. we were all to meet at the Baroness Burdett-Coutts's to sign the agreement. At 3 p.m. we left Stratton Street and went to our different homes to pack up, as we were all to be at Paddington Station at 7.30 p.m."

**THE OLD STYLE.**—Mrs. J. M. Flower, the vice-president of the Illinois Training School for Nurses, founded in 1880, writes thus of the nurses who worked in the Illinois Hospital before that date: "The attendants at the hospital at this time were mostly persons appointed, not for fitness, but as a reward for the services of some ward politician, and being sure of their positions, no matter what work they did in the hospital, as a general rule did nothing, and there being no supervision over them in the wards the neglect of the patients was terrible. If we were to publish to-day the testimony of physicians and others cognizant of these things taken at the time, it would hardly be believed that such abuses could exist, though they were probably no more than those of other city hospitals exclusively under political control. We have before us a letter from the superintendent of the Philadelphia Hospital, in reply to one asking about a nurse formerly employed there, in which he says: 'As the nurses at that time (before the Training School took charge) were all either women of bad character, or convalescent patients, I can only infer a nurse here at that time would not be desirable.'"

**WOMEN'S WORK.**—When a tentative effort has resolved itself into an accomplished fact, though we may all admire and recognise its success, we are apt to forget the struggle which the first effort must have cost the pioneers, and to think of the fact as having always run on the same "oiled wheels" on which it goes at present. For instance we see women working everywhere, as doctors, nurses, lecturers, journalists, clerks, etc., and their labour is recognised as something really marketable. Perhaps sometimes, when these women get disheartened and weary over their work, it would be a help to them just to think what the women went through who first resolved to see how their brains and education could manage the work usually allotted to men. The struggle at the outset must have been far more wearying, when women were scarcely considered competent workers, than it is now, when the presence everywhere of female workers shows that prejudice is conquered. Lady doctors gratefully remember Dr. Elizabeth Blackwell and Dr. Garrett Anderson. Nurses are ever mindful of those who opened up the noblest women's work which exists, and so with all the other workers, they will do well to remember the efforts of the first energetic few. We lately heard "the happy old maid" described as a "product of the nineteenth century." That is just what we believe her to be, just because she need never want something to do, and certain it is that it is generally lack of occupation which makes an unhappy woman.

**AN APPEAL TO NURSES.**—A patient lately went to a hospital to be examined; he was sent up from the out-patient department to a ward, as it was desirable he should go to bed and wait for a thorough examination which would necessarily be a long one. The man was very ill, he was a nuisance in the ward; he was an irregularity in a vast system which went on wheels while all was straight. The nurses were not glad to see him, and unfortunately they forgot to hide it; they were also so forgetful they never offered him a cup of tea or any sustenance, though he told them he had left home early in the day. The examination over, the physician, as kindly as possible, told the man the case was hopeless, he could only offer him a bed to die on; the physician knew the man was penniless. To his surprise the man refused the offer of the bed, saying simply, "I prefer to starve, sir." He gave no reason for this statement and went his way. When the end came he was tenderly nursed by a district nurse; then in a moment of impulse he spoke to the parish doctor, and told him how the heartlessness of his welcome to the hospital had struck him so forcibly he felt it impossible to remain there. The nurse had said, "What a nuisance!" when he handed her the doctor's note. Only a careless phrase of a woman who was doubtless an excellent nurse, but when will our nurses remember that to them no careless speech is permissible; that sympathy and gentleness must be with them unfailing? Often and often this failure to welcome a patient properly is at the root of many a hospital scandal. It is so easy to forget that the sickness and sorrow, which is an everyday fact to the nurse, is a very personal thing to each patient. That man, come to hear his death-sentence, ill and weary, was treated not only carelessly, but cruelly by those nurses who failed to welcome and to minister to him. Greater guard over the tongue is required by nurses who are apt to forget the human being in the patient.

**ASYLUM ATTENDANTS.**—The Medico-Psychological Association at its autumn meeting at Glasgow discussed at length a scheme for the better training of asylum attendants. The very best qualities of a nurse are required for mental nursing, and what the Medico-Psychological Association is now aiming at is the production of these qualities out of the attendants and nurses who come into the asylum service. It is a speciality as much as obstetric nursing, but it should never be divorced from nursing proper or left out in the cold as a less important charity than general nursing. Under the new regulations a system of training will be instituted extending over two years, a period of three months' probation being required before training is formally entered on. The scheme of training will embrace the study of handbooks prescribed for the purpose,* exercises under the head and ward nurses, clinical instruction by the medical staff, and lectures and demonstrations. The scope of training will comprise the ordinary requirements of general nursing, instruction in the general features of mental disease, the management of mental cases, and how to render first aid. At the end of training, candidates recommended by the medical superintendent will come up for examination, and after giving satisfactory evidence of proficiency will receive the certificate of the Association. The scheme is optional for asylums; but there can be no doubt that the majority of asylum medical officers will undertake it.

* "Handbook for Instruction of Attendants on the Insane." Prepared by a Committee of the Medico-Psychological Association. (London: Messrs. Baillière, Tindall and Cox.)

### Sketches of Insanity.

By Francis H. Walmsley, M.D. (Senior Assistant Medical Officer of Leavesden Asylum).

### X.—EPILEPTIC INSANITY.

### I.

Epilepsy is one of the most dreadful and appalling diseases that afflicts mankind; so terrible indeed is the spectacle that, in the earlier ages, the only interpretation that seemed capable of accounting for the condition into which the unfortunate sufferer was suddenly plunged was the belief that some unclean spirit or torturing fiend had entered into and taken possession of him.

In the graver form of epilepsy the person attacked—while engaged in his ordinary pursuits, and in apparent health—suddenly utters a peculiarly piercing and startling scream, and instantaneously loses consciousness, with terrific force he is struck down, his face coming into violent contact with the ground, the whole body is thrown into a state of muscular rigidity—becomes tense and stiffened out—the muscles can be felt like bars of iron, the walls of the chest are perfectly fixed and motionless, the breathing is completely arrested, a death-like pallor overspreads the countenance, the patient appearing as if about to expire. This stage lasts for a few seconds only; the face then becomes flushed and the veins of the head and neck distended, the redness rapidly changing to a livid dusky hue, the features are hideously distorted, the eyes wide open, the pupils dilated, the eye-balls roll convulsively upwards or inwards, the lips are blue, the tongue, swollen and purple, and often deeply bitten, is protruded through the half-opened jaws—suffocation seems imminent. This stage lasts from ten to forty seconds at most, then commence convulsive movements of the body and limbs, which vary from the most trifling and transitory spasms to the most frightful and long-continued struggles. The temperature of the body rises, the skin becomes covered with perspiration, and saliva, reddened with blood, or not, issues from the mouth. When the paroxysm has reached its crisis the muscles relax, the convulsions subside, the lividity disappears, the breathing becomes more tranquil and the countenance more natural; this stage lasts from half a-minute to two minutes at the most, so that the whole duration of the attack varies from two to three minutes, and in many cases still less than this.

After the paroxysm the patient looks like an individual who is stupified by drink; for a length of time, varying from a few minutes to half an hour, he remains in a condition of profound stupor and complete immobility; at length he opens his eyes, looks around him in a perplexed or frightened manner, and falters out a few unintelligible words; on regaining consciousness the patient either rapidly recovers or remains confused and bewildered for some hours, or it may be a day or two before complete recovery takes place.

The epileptic paroxysm varies in intensity, in violence, and in suddenness of seizure; in mild cases only one limb is convulsed, in others only the face, the lip, or the eye. Frequently one half of the body is affected, but in the severer form the muscles of the face, trunk, and limbs are violently convulsed.

Very often the patient suffers severe injuries; the face may be bruised and lacerated, he may fall into the fire and be frightfully burnt; the convulsive movements may be so severe as to produce dislocation of the jaw or the arm; again, after the attack, the patient may remain temporarily paralysed on one half of the body.

There are singular disturbances of the nervous system known as warnings, which sometimes usher in epileptic seizures, a peculiar sensation which the individual compares to a kind of wind, or of vapour, or to tingling starts from some portion of his body, as the hand or the arm, and rapidly spreading up to the head the fit begins; this sensation is transient, and lasts from one second only to a minute sometimes. Again, prior to the attack, the patient may manifest change of temper, indicated by extreme irascibility; he may complain of headache, giddiness, palpitation, cramp, trembling; he may display a tendency to run forwards or backwards, to turn somersaults, or to roll round and round on the ground; or he may perceive a peculiar smell, or hear strange sounds or voices, or he sees before his eyes, animals, departed friends, witches, devils, or experiences a sudden horror or trouble.

Frequently epilepsy manifests itself in the form known as epileptic vertigo or the "small seizure," though in this variety there is an absence of the violent convulsions, and the symptoms generally are less formidable. Nevertheless, it is a very grave disease.

Epileptic vertigo is characterised by transient strange phenomena which sometimes consist only in giddiness, in a sort of astonishment, in ecstasy, or a fit of absence, there may be little more than a momentary interruption to the continuity of the patient's thoughts; if he be closely observed at this moment, his pupils will probably be seen to dilate, his face to become momentarily pale, the eyes fixed, a few convulsive twitches may play over the face, there may be a totter of step, yet he does not fall; if he has an object in his hands, he drops, or convulsively throws it away from him; the whole lasts from two to four seconds and sometimes more, the attack is then over, the patient recovers himself completely, resumes his occupation or the conversation in which he was engaged; if seized while walking, he may continue his movement as regularly as before the fit.

An individual subject to epileptic vertigo may, while playing at cards, and holding in his hand a card which he is about to throw down, suddenly become motionless, shut his eyes or stare before him, and then, after drawing a deep sigh, he may continue to play.

Again a musician seized with one of these transient attacks while playing the violin, strange to say goes on playing during the attack, and neither hears nor sees those he is accompanying, he still plays in tune, and performs without a fault the musical phrase which he had read just as his mind became effected.

Again an architect, long subject to epileptic vertigo, does not fear to go up the highest scaffolding, and yet he is perfectly aware that he has often had fits while walking across narrow planks, at a pretty considerable height; he has never met with an accident, although when in a fit he runs rapidly over the scaffoldings, uttering, or rather shrieking out his own name in a loud abrupt voice, a quarter of a minute afterwards he resumes his occupation and gives his orders to the workmen, but unless he be told of it, he has no idea of the singular act which he has been committing.

Epilepsy occurs far more frequently between the ages of ten and twenty than at any other period of life, though no age is exempt from attack.

Very often, in the beginning especially, the seizures take place during the night, frequently at the moment of going to sleep or at the moment of waking. An individual may thus be afflicted for eight or ten years, although nobody, not even himself, suspects the existence of his dreadful disease. Three very remarkable men—Cæsar, Mahomet, and Napoleon are said to have suffered from epilepsy. If they did, it must have been in a mild and infrequent form, for there was no indication in any one of them of any loss of brain power, or any tendency to general paralysis. Perhaps other diseases were more likely to be confounded with epilepsy in those earlier periods than they are now. In any case, there is much room for doubt in all three instances.

(*To be continued.*)

### A Turn of the Wheel.

"BLANKSHIRE.—District nurse wanted for a healthy village. Liberal terms to a suitable lady. Full particulars to Mrs. F., Manor House, Cotesby."

"How would that do, Alice?" said my cousin, with one of her sweet little tinkling laughs. There was that about Conny, no amount of this world's roughest tear and wear managed to dry up the fresh springs of her laughter, which bubbled up irrepressibly.

Poor, winsome little Conny! She and I had travelled thorny paths since the old days down at Branksome, when we were Granny's spoiled charges, and, as everyone supposed, Granny's heiresses. It was not until our girlhood was merging into womanhood that adversity's storms broke over our heedless heads. Granny died; and we had scarcely recovered the stupefaction over our first life-sorrow, when we found ourselves to be beggars. Not yet is it quite clear how it came about, but, ignorant as we girls were, we were conscious of cheatery and swindling, which had, possibly, been going on for some time. Out into the wide, unknown world Conny and I went forth, to fight our way among the army of bread-winners.

Thus it came to pass in course of time that I, a trained hospital nurse, wearied and battered by exceptionally hard town work, was intently studying the advertisement page of our own little weekly in search of some fitting opening in the fresh, dewy country to revive me. Conny, who slaved, late and early, giving music lessons to anybodies and everybodies, at a shilling an hour, hung over my shoulder, her dear curly fair head resting against my own. It was a day off, and I was spending it in Conny's room—a queer little room up in the clouds, but a dainty one, for poor Conny was a gentlewoman; nothing vulgar, nothing showy could there be in her surroundings.

"Do you *think* it would be any use for me to apply?" I asked doubtfully.

"Apply! Of course. You'll write this very hour. Who knows, Alice, this may be the gate opening out to fortune for you—for us both!" and Conny laughed again.

"Oh, child, what a human cork you are!" I exclaimed, half fretfully; "don't you see, if I were successful in getting this appointment"—and I touched the advertisement—"we should be parted, you and I, little Con," and the tears rushed to my eyes.

"Parted!" I could feel the sudden upward sweep of Conny's long eyelashes against my cheek; "Never, Alice! If you do go to this healthy village—and what they want you for, if it be healthy, is more than I can imagine—I go also. I'll pack up my traps and come down. We'll have a room between us, and I'll teach healthy villagers music and singing for next to nothing. I feel a mission rushing over me to develop music in the rustic soul. Think of the poor dears in their dumb helplessness. No words, no sounds have they to tell out when they are sorry or when they are glad. Nothing but hymns! Oh, yes, my dear, there's no doubt we have found our vocations. Write, write!" and the materials were impulsively pushed before me.

As all great events do, it came to pass, with bewildering swiftness. Before a month had flown over our heads Conny and I were established in a honeysuckle-covered cottage of the "healthy village." In answer to my application, my testimonials were demanded by Mrs. Folliot, of the Manor House, at Cotesby, and were promptly and heartily supplied from the hospital where I had trained.

I was chosen at once out of many others, and with, as I have said, bewildering speediness Conny and I found ourselves in what she called our new diggings. We had been received with warm-hearted welcomes by the good soul, in whose cottage we were to be quartered; we had been interviewed by the clergyman of the parish, a young man and shy, who seemed uneasy and appalled at our ridiculously youthful appearance; but we had seen nothing as yet of the Manor House, nor its mistress.

"'Tain't surprisin' that," vouchsafed our rubicund landlady. "Mrs. Folliot, she be main queer. Lor, but what could 'un expect from she? Her ain't none o' the quality—the real gentry that is," confidentially added Mrs. Skeat.

"She be but a poor thing, wi' trumpery health," she went on in desperation, seeing neither Conny nor I meant to help her out by questions. "Always ailin' and shakin' with narves. Ye see, she ain't barn to greatness; she wor only Lawyer Folliot's wife. He made a rare few pickings in business, did Lawyer Folliot, honestly or dishonestly, that's as may be. Then he bought the old Manor House; some say he helped hisself to it, wi'out buying of it, but I don't never gossip, not I; I couldn't abide to. Only the lawyer, he didn't live to enjy his ill-gotten lands; maybe the feelin' that he'd cheated the old fam'ly choked he—they did say as it were apperplexy took he off. And there's she, lone and ailin' in the big house, with a swarm o' sarvants that's she's afraid of, and nary a friend." By this time the dame, who had been backing out of our presence, bearing the tea-tray aloft, came in collision with the door, a catastrophe that speedily relieved us of her presence.

Lawyer Folliot! Conny and I sat staring mutely at each other.

"It must be simply a coincidence of name," I murmured at last, with a determined shake of my shoulders.

Folliot was the name of Granny's lawyer, who had never, to our clear satisfaction, given us to understand why everything, home and money, had crumbled away at Granny's death.

"Oh, no doubt! A brother, perhaps!" and, with a relieved laugh, Conny was off to the old-fashioned garden to tease the solemn tortoise as that antiquated dignitary heaved himself along the flower-bordered path.

"You are the District Nurse?" It was a tremulous voice, and came, half-muffled, from a heap of frilled cushions, which well-nigh buried the shrunken form, huddled in a huge, deep chair.

The room was so dim, for the blinds were drawn far down, and the windows shut tightly, that I could barely see the pallid features of Mrs. Folliot until my eyes became accustomed to the darkness.

"I hope you'll like the village—and be comfortable," went on the voice, monotonously. "Our clergyman—he's new and energetic—never gave me any peace until I advertised for a parish nurse, for the village poor. Not that I was unwilling, for God knows all I'm anxious for is to do some good with Joseph's money—to make up, if I can!" The last words ended in a low whimper.

It scarcely needed my professional knowledge to detect that the mistress of the Manor House was in a deplorably broken-down state of nervous exhaustion. She was a difficult subject, but, by dint of extreme gentleness and caution on my part she ceased to tremble; she even grew communicative, putting me in possession of her entire history—a most unhappy one. Her marriage had been an affair made up for the good of her family, and she had been hustled into giving up her lover for that end. With no love to blind them, her eyes were open to Lawyer Folliot's systematic and wholesale cheateries; the illgotten riches weighed heavily upon her. Now that he was dead, and the burdensome wealth in her own hands, she loathed and feared it still more.

It was not my last visit to the Manor House; and, as time went on, I was sent for, every day, at first, it seemed for my professional services; but, by degrees, Mrs. Folliot grew to depend upon me, almost childishly, in all matters. Then Conny, too, had to be brought to amuse the despondent woman. Poor Conny, who drooped sadly over the lack of pupils, harassing herself because of her inability to bring grist to the mill. Thus it was in that way it came to pass Mrs. Folliot learned, from Conny's lamentations, our pitiful story.

Coming in upon the two of them, one day, from my first "case" in the healthy village—that of a scalded wee cottager —I found Mrs. Folliot in a state of agitation bordering on frenzy.

"I've found you out!" she cried, wringing her hands; "you are the hapless orphans Joseph turned out of Branksome, and cheated of their money. I knew he did! And I told him the curse of the Almighty would blight him for such ill-doing, and surely it did. Just when he'd got all this in his clutch death swept him off. He was a bad man, God forgive me for saying so of the dead!"

It was all true enough, and from that day forth Conny and I were taken into the Manor House, and into its remorseful owner's heart. In "making it up" to us, as she called it, Mrs. Folliot's mind calmed into placidity, and health of body as a consequence ensued. It was a strange turn of fortune's wheel that made me the petted daughter of Lawyer Folliot's house, for so I am this day.

But Conny! Conny went back to the village, where she is giving music and singing lessons to the choir boys, to the unbounded admiration and satisfaction of the shy young clergyman—whose wife she is.

### Everybody's Opinion.

[*Correspondence on all subjects is invited, but we cannot in any way be responsible for the opinions expressed by our correspondents. No communications can be entertained if the name and address of the correspondent is not given, or unless one side of the paper only be written on.*]

### "TRAINED MIDWIVES."

"One Who Knows the Rural Poor" writes: "I will tell you why trained midwives are not in more request in country districts. They are, as a rule, unmarried women, and the poor have a very strong prejudice—as well indeed as many far above them in education and position—in favour of a married woman. I have heard many say, ''Tain't a right thing for a woman to do as ain't had children of her own,' and I am bound to say that I fully understand their view. Does it not strike you as somewhat revolting to send a comparatively very young woman to attend such cases? I know one very sensible wife of a clergyman, and the mother of a well-grown family, who says, 'Such women may perform the mechanical duties required by their sex, but into the sacred spiritual duties of the office, none but the wife and mother can possibly enter.' That is why the poor prefer the rough, and ignorant even, methods of their own *sage femme*."

### HOSPITAL NURSES.

"A Lady" writes: Few people seem to think anything of nurses belonging to the accident hospitals. They say, "Oh, there is no nursing required; just bind up their wounds, or set a leg, and nothing more is to be done; no nursing, no watching." But I, as one of the "accident nurses," can contradict that. If a poor man is brought in nearly scalded to death, perhaps delirious, and cannot in any way help himself, is there no nursing required in that case? Take another instance. A boy is brought in crushed, perhaps a heavy weight has fallen on him, or he is crushed between a train. When brought in he is so bad that even the surgeons think he cannot possibly live to get him in bed. If in a fortnight that boy is so far recovered as to sit up a little, and then in a few days walk about the ward, has that been through careful nursing or not? I think all those who say there is no nursing required in "accident hospitals" should make it a point of duty to go and visit those places, and see the grand work that is being done; then they can express their opinion afterwards. I hope the day is not far off when "accident nurses" will receive their just due from the public so greatly benefited, and that those who care to may have the opportunity of learning all classes of nursing, even accident work.

### Notes and Queries.

To Correspondents.—1. Questions may be written on post-cards. 2. Advertisements in disguise are inadmissible. 3. In answering a query please quote the number. 4. If a private answer is desired, a stamped addressed envelope must be forwarded.

Notice.—We must really ask our correspondents to be more particular in enclosing name and address. Constantly we receive queries or letters with no name attached.

### Queries.

(34) Can some one kindly let "Vanessa" know of some children's hospital for which she could do some work; also what article of clothing would be the most useful for her to make for a children's hospital?

(35) *Epilepsy.*—Can any reader recommend a simple book on epilepsy and its treatment, and state cost of the book?

(36) *Nursing Abroad.*—A lady wishes for information about hospital nursing abroad; more particularly as to how to set about getting into a hospital in South Africa, India, or Cyprus.

### Vacancies.

The following vacancies are announced:

### Nurses.

Birmingham Eye Hospital; £25.

Bournemouth Institute; £25.

Borough Hospital, Birkenhead.

Blackheath and Richmond Institutions.

Birkdale Home, Southport.

Brixton Institute.

Bromley and Surbiton Institutions.

Children's Hospital, Pendlebury; £20.

Cheltenham Institution.

Cambridge Home.

Chelsea Infirmary; £18.

Hanover Institute, W.

Hartlepool Union; £25.

Hampstead Home Hospital Institute; £20.

Levick Institute, Paris.

Llanelly Hospital; £25.

Liverpool City Hospital; £25.

Lowestoft Association; £26.

Nursing Institution, Wimpole Street.

Nurses' Home, Hull.

Nurses' Home, Frome; £22.

Poplar Union, £20.

Royal Hospital, Belfast; £25.

Rotherham Hospital; £16.

St. Helena Home, N.W.

St. Saviour's Infirmary, S.E.; £20.

St. John's Home, West Kirby; £24.

South-Eastern Fever Hospital, New Cross; £30.

Victoria Home, Bournemouth; £25.

Workhouse Infirmary Association.

### Probationers.

Bedford Infirmary.

Dewsbury Infirmary.

Jessop Hospital, Sheffield.

St. Helena Home, N.W.

St. Mary's Day Nursery, Plaistow.

### Matron.

Brighton Borough Hospital; £60.

### Sisters.

Leeds Fever Hospital; £32.

Royal Albert Hospital, Devonport; £25.

Seamen's Hospital Society; £30.

Victoria Infirmary, Glasgow; £24.

### Amusements and Relaxation.

### SECOND QUARTERLY WORD COMPETITION.

Commenced July 5th, 1890, ends Sept. 27th, 1890.

Three prizes of 15s., 10s., 5s., will be given for the largest number of words derived from the words set for dissection.

Proper names, abbreviations, foreign words, words of less than four letters, and repetitions are barred; plurals, and past and present participles of verbs, are allowed. Nuttall's *Standard* dictionary only to be used.

N.B.—Word dissections must be sent in WEEKLY not later than the first post on Thursday to the *Prize Editor*, 140, Strand, W.C., arranged alphabetically, with correct total affixed.

The word for dissection for this, the ELEVENTH week of the quarter, being

"BARLEY."

| Names. | Sept. 6th. | Totals. | Names. | Sept. 6th. | Totals. |
|---|---|---|---|---|---|
| Dulcamara | 28 | 508 | Jenny Wren | 29 | 514 |
| Tinie | 29 | 553 | Rhoda | — | 242 |
| Henri | — | 395 | Annabel | — | 17 |
| Agamemnon | 30 | 563 | Spare Moments | — | 19 |
| Patience | 29 | 554 | Qu'appelle | 29 | 522 |
| Brisbane | — | 332 | Nurse | — | 29 |
| Nurse Hilda | 30 | 535 | A. B. C. | — | 251 |
| Lightowlers | 29 | 548 | Brickbat | — | 77 |

### Notice to Correspondents.

N.B.—Each paper must be signed by the author with his or her real name and address. A *nom de plume* may be added if the writer does not desire to be referred to by us by his real name. In the case of all prize-winners, however, the real name and address will be published.

Competitors can enter for *all* quarterly competitions, but no competitor may take more than *one first prize* during the year.

